# The Judge's Report

**Published:** 1918-12-14

**Authors:** 


### "THE HOSPITAL" LEAFLET COMPETITION.

### THE JUDGE'S REPORT.

OUT of the fifty-two leaflets sent in, twelve reached a certain standard of excellence in style and matter. These are all dealt with below. We may add here that each one of them is clearly expressed, gives a succinct account of the necessary training and how to obtain it, develops the moral and material advantages of the work, and gives sufficient details concerning the duties to be undertaken which will help to enlighten women about the work before offering themselves as candidates.

### Names of Prize Winners.

The leaflet selected for the *First Prize* of £3 3s., "Public Health Work for Women," by Miss Anita Robinson, is likely to be particularly useful to women choosing a career. It discusses the various branches of "Public Health Work" open to nurses, the uses of the various certificates which they must obtain in order to qualify for certain posts, and the value of this career to the individual and to the public. It is hardly too much to say that it embodies information nowhere else so clearly outlined in so small a compass.

"Factory Nursing," by Miss C. U. Kerr, to whom we award the *Second Prize* (£2 2s.), is an interesting description of this new branch of nursing. It explains the best form of training to prepare for it, gives an outline of the day's work, mentions the posts to which it leads on, and leaves the reader thoroughly convinced of its attractions.

"District Nursing," by Miss M. Wiles, is the best of many good leaflets on the same subject, Miss J. G. Gilchrist's leaflet on "School Nursing" is equally good, and the *Third Prize* of £1 1s. is therefore divided between these two competitors. The information Miss Wiles' leaflet imparts is exactly what the candidate requires. It is candid in enumerating the hardships of the life, yet the true spirit of vocation breathes in it, and it expresses the conviction that no woman worth her salt is deterred from a career of value to the community by learning that it will make demands on her physical, mental, and moral powers. Miss J. G. Gilchrist's paper is an enthusiastic description of the school nurse's life, written with charm and individuality. The information is sound and the conclusions reached are convincing.

### Highly Commended.

Three other leaflets on "District Nursing," by Mrs. M. M. Carnegie, Miss K. O'Sullivan, and Miss Barbara J. Forbes, are highly commended. Among the highly commended also are "The Hospital Sister," by L. W., a really delightful appreciation of the ward sister's life and work; and "Mental Nursing," by Miss M. D. Laurence, bringing into relief the interest of overcoming the admitted difficulties of the work and the cheering recreations by which its strain is alleviated. Two leaflets by Miss F. E. Evelyne Harris, one on "Massage" and the other on "Nursing in a Nerve Hospital," are also highly commended. They are written with the zest of one who has proved the life she describes, and has found it good, independently of worldly advantages. One more leaflet must be commended. It is "A Plea for Infirmary Nursing," by N. J. Phelp. It is written in homely style to suit the comprehension of a girl hesitating whether or no to apply for training. It carries her through all the early stages about which candidates have so many misgivings, and leaves her looking back on the time spent in training as among the happiest years of her life.

### Honourable Mention.

We cannot forbear saying something of other leaflets which have given evidence of sincerity, sound knowledge, originality, and the spirit of vocation. Without exception writers have reflected the glow of their conviction that nursing is by far the best career for a woman, and moreover that the branch of it they know offers all that a woman can desire for the satisfaction of a true purpose in life. Those deserving honourable mention are Miss Adams, who sends two good leaflets, one on "Midwifery," practical and welcome, and the other on "Fever Training," which contains a pleasant account of life in a fever hospital. Miss Frances E. Farley writes a eulogy of the life of a Cottage Hospital matron which is well put together and sound in ideals. Miss van Crane sends an attractive essay on "District Nursing," while Miss Maud Lewis, on the same subject, gives a good description of the attractiveness of village life and its many opportunities of doing good. Miss Hairs is quite good on "The Nursing of Sick Children," advising the children's nurse to take three years first in this branch of work and subsequently three years of general training. Miss Balderstone infringes the title of a book in writing in homely and familiar style for the timid and

**"The Hospital" Leaflet Competition—(*continued*).**

hesitating probationer. Nurse E. May Palmer has cleverly cast her leaflet into the form of a letter of advice to a widow who wants to begin nursing. The reasons set forth for taking up village nursing with midwifery under these circumstances are well put, and advice to a younger woman to take a full three years' training is adroitly introduced. Miss Townend has also a good leaflet on "Village Nursing as a Career for Women." Sister N. Edmonds, in her rather indefinite leaflet on "Medical and Surgical Work" (a subject on which too few papers have been received), has some nice things about the relations of a nurse with patients dreading operation. Two otherwise good papers on "Private Nursing," by Florence Nightingale and E. Nightingale, are vitiated by the advice given to take *two* or three years' general training. Both, however, urge the nurse to qualify in several directions. Miss Begg, on "The Nursing of Chronic Patients," is very sound and sensible. Miss Elinor Shaw has a good paper on "School Nursing, and so has L. K. B., who writes in familiar style and conveys a vivid impression of the duties which lie before the nurse in this branch of work. Our one regret is that there are not more leaflets on the institution side of nursing.

